# Management of Superficial Abscesses: Scope for Day Case Surgery

**DOI:** 10.1155/2014/308270

**Published:** 2014-05-26

**Authors:** Martha Nixon, Jacob A. Akoh

**Affiliations:** Department of Surgery, Derriford Hospital, Plymouth Hospitals NHS Trust, Plymouth PL6 8DH, UK

## Abstract

*Background.* Patients presenting with superficial abscesses are often regarded as low priority and given a less efficient service. *Aim.* The aim of this study was to investigate the efficiency of emergency treatment of superficial abscesses and to identify areas for service improvement. *Method.* A retrospective case review of patients admitted to Derriford Hospital, Plymouth, over a four-month period. *Results.* Ninety-seven patients were included in the study. Seventy two (74%) arrived between 08.00 and 16.00 hours. Overall, 75 patients (77%) were referred on weekdays with 22 patients (23%) during weekends. Seventy-two patients (74%) had treatment under a general anaesthetic. Sixty-three percent of operations occurred within the working day. The time interval between admission and surgery ranged from 52 minutes to 38 hours (mean ± SD 16 ± 9.15). The length of admission ranged from 5.3 hours to 11 days (mean 36 hours). Of the one hundred overnight beds used by the 97 patients, 30 nights were spent awaiting surgery and 70 following surgery and awaiting discharge. *Conclusion.* Eighty-nine percent of the patients would have been suitable for treatment as day cases. This review shows that a simple service redesign has the potential of reducing inpatient bed occupancy and improving the patient's journey.

## 1. Background

Abscesses form a significant part of the emergency surgical work in many units. They are generally regarded as lower priority than other surgical emergencies, meaning that the definitive treatment for patients presenting with superficial abscesses is often delayed. This is inconvenient for patients and worsens the sickness absence statistics for employers. There may also be an unnecessary burden on an overstretched health service whilst patients occupy beds awaiting surgery [1]. The aim of this study was to investigate the efficiency of the management of superficial abscesses and identify areas for service improvement such as the provision of a day case abscess service.

## 2. Patients and Methods

This is a retrospective case review of all patients with superficial abscesses admitted to the Surgical Assessment Unit, Derriford Hospital, under the care of general surgeons between July 2011 and November 2011. The list of patients was generated using the hospital coding system. Superficial abscesses included perianal, pilonidal, buttock, chest wall, abdominal wall, back, groin, axillary, upper limb, and lower limb abscesses. There were 2789 patients admitted to the Surgical Assessment Unit (SAU) over the four-month study period. Of the 117 patients identified who were admitted with superficial abscesses, 20 were excluded due to incorrect coding, unavailability of notes, or incomplete notes. Information was gained from clerking proformas, daily entries, operation notes, anaesthetic charts, drug charts, and discharge summaries. Descriptive analysis of day and time of admission, period from admission to surgery, type of anaesthesia, and length of stay was performed. The reasons for bed occupancy were also recorded.

## 3. Results

Of the 97 patients included in the study, 58 (60%) were male and 39 (40%) were female. The mean ± standard deviation (SD) age was 38 ± 16 years (range 16 to 87 years, median 34 years). Seventy-five percent of patients were referred by their general practitioners while 24% came from the emergency department with the remaining arriving from oncology outpatients.

**Figure 1:**
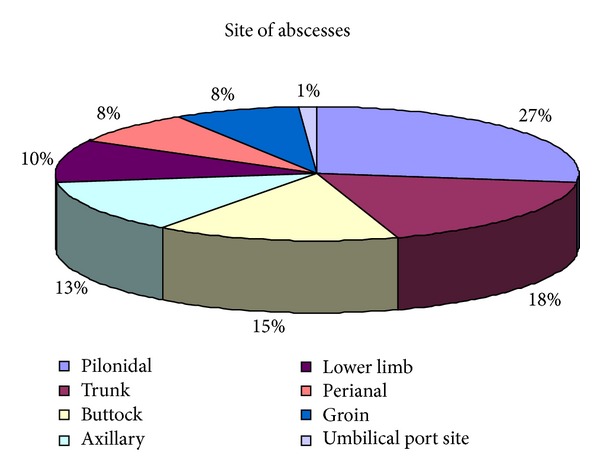
Site of superficial abscesses in 97 patients.

**Figure 2:**
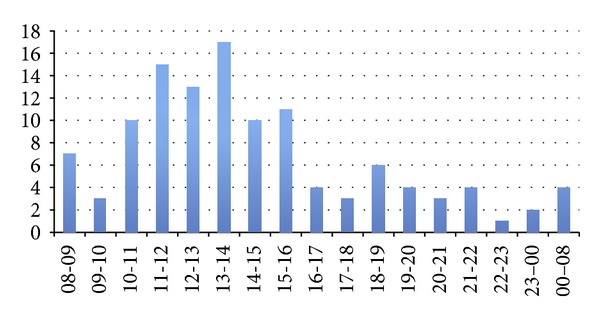
Time of admission.

**Figure 3:**
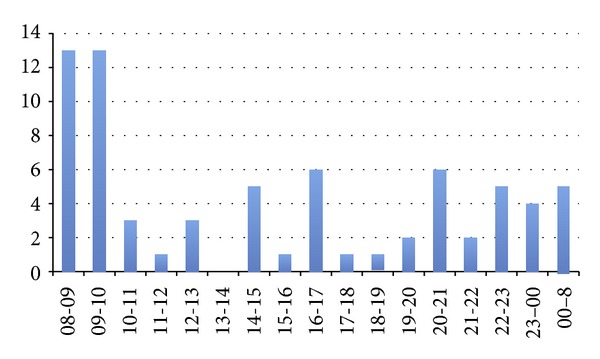
Time of operation.

The site/type of abscesses is shown in Figure 1 with pilonidal abscesses, the most common type accounting for 27%. The number of admissions for each day ranged from 0 to 5 with 74% arriving between 08.00 and 16.00 hours (Figure 2). There were no admissions for abscesses on 23% of the weekdays. The majority of patients underwent incision and drainage under a general anaesthetic (GA) (74%). Thirteen percent of patients had an incision and drainage under local anaesthetic (LA) and 13% of patients did not require surgical intervention and were discharged with conservative treatment only. Most procedures were performed between 08.00 and 10.00 hours (Figure 3); 63% of patients had the operation within working hours (08.00–17.00). The time interval between admission and surgery ranged from 52 minutes to 38 hours with a mean ± SD of 16 ± 9.15 hours.

**Figure 4:**
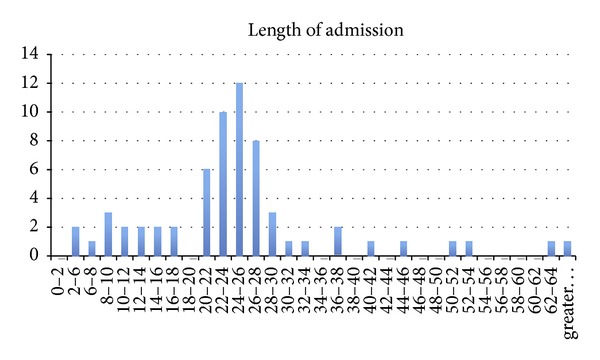
Length of hospital stay (hours) for 97 patients with superficial abscesses.

The length of admission ranged from 5.3 hours to 11 days with a mean admission time of 36 hours and mode of 24–26 hours (Figure 4). Nineteen patients were given overnight leave to return at 07.30 hours the following day for surgery. Twenty-seven percent of patients undergoing surgery before midday stayed overnight awaiting discharge. Review of the actual figures for bed occupancy showed that a total of 100 beds were occupied overnight. Thirty bed nights were spent awaiting surgery whereas 70 were spent following surgery and awaiting discharge. Fourteen of these nights were spent as inpatients as the patients were receiving ongoing medical treatment, mainly antibiotics. The reasons for antibiotic use and necessity were beyond the scope of this study.

## 4. Discussion

The treatment of superficial abscesses represented approximately 4% of the emergency surgical workload over the four-month period. Superficial abscesses in appropriate areas of the body are amenable to topical analgesic/anaesthetic agents [2, 3] but the majority require GA to allow adequate treatment of what is a painful condition [4]. In this series of abscesses referred to the SAU, 74 patients (76%) were treated with an incision and drainage under GA. The apparently high proportion might have been due to the fact that patients presenting with relatively simpler forms of superficial abscesses were drained in the emergency department and discharged home leaving the more complex cases to be seen in the SAU. However, this number might have been higher if the treatment under general anaesthesia was a more efficient service. In the process of obtaining an informed consent, patients become aware of the potential time delay whilst waiting for an incision and drainage under GA compared to treatment in the ward under LA. It is reasonable to assume that some patients might have opted for incision and drainage under local anaesthesia in order to achieve a shorter period of hospital stay. If the treatment time scales of patients undergoing surgery under GA or LA were not unreasonably dissimilar, it would be conceivable that more patients might decide to undergo incision and drainage under GA.

There was an average delay of 16 hours to surgery and an average length of admission of 36 hours despite 42% having their procedures before midday. These patients were young (mean age: 38 years) and healthy and in most cases required only a less-than-30-minute operation. The number of patients detained in hospital due to the lack of an adult companion at home on the day of surgery was not known. Only 11% of patients in this study required further treatment in the form of antibiotics or dressing care in the hospital after the operation. Eighty-nine percent of patients would therefore be suitable for treatment as day case procedures.

Given appropriate service redesign, it would have been possible during the study period to avoid the use of 86 beds overnight. With an efficient and streamlined day surgery service, this can be extrapolated to approximately 258 beds saved per year. This would save around *£*77,000.00 for our NHS Trust alone. It would also free up beds for elective surgery or more appropriate nonelective cases. Patient satisfaction would be improved by a more convenient service.

The current management of abscesses has been shown to be variable [5], but in our experience most abscesses have traditionally been treated with incision and drainage and packing to allow healing by secondary intention. In certain areas, such as breast abscesses, it is now routine practice to perform aspirations rather than incision and drainage [2, 6, 7]. This practice could be applied to abscesses in other sites. There is evidence to show that abscesses elsewhere can be successfully treated with much smaller incisions and healing by primary closure [8–11]. Adopting this practice would mean that many more abscesses can be treated with local anaesthetic and therefore would not require hospital admission. This would also reduce costs in the community caused by regular packing and be more convenient for patients [12]. There is evidence that packing abscesses although practised widely may interfere with wound healing and is no longer recommended for most abscesses [13]. A new approach to the management of abscesses combined with a more flexible service redesign may eliminate the current problems highlighted in this study.

This study has important limitations. The size and severity of abscesses were not analysed and reasons for delayed access to theatre were not specifically identified. Furthermore, the choice of the type of anaesthesia involved a selection bias due to the significant differences in hospital stay between those having surgery under LA and GA. The average length of stay for those undergoing incision and drainage under LA was 8 hrs and 43 minutes. Despite the foregoing, the numbers and pattern of admission to this trust would make a day case service a feasible option. This could be run Tuesday–Friday with 2-3 cases per day. This could be added onto the end of the elective day case list so that the patients referred during the day would all be available for surgery on the same day. An alternative approach would be to allow all patients (excluding those with systemic effects of sepsis or severe pain) to be clerked and released to go home on overnight leave with instruction to report for surgery the following morning. Their abscesses could be drained in theatre while the duty consultant conducted their posttake ward round. The drawback of this approach is that on occasion it may remove the specialist registrar from the ward round or even lead to delay in starting surgery on more urgent or complex cases. A third option might be to utilise theatres finishing their elective lists early to do an extra abscess or two. A pragmatic solution may be to combine all three options to ensure day case management of patients with superficial abscesses. A few patients (11% in this study) would require admission from the day case unit for ongoing treatment with antibiotics if there was sufficient sepsis or cellulitis to warrant it. Other studies have also shown that huge savings can be made, with no harm to patients in other domains of general surgery [14–18].

## 5. Conclusions

In the face of an ever increasing demand on the National Health Service, innovative ways of improving the service are required. This should be aimed at streamlining a more efficient service and also improving the patient's journey. This review of a common nonelective surgical problem shows that a simple service redesign may reduce inpatient bed occupancy and improve the patient's journey. Further analysis after applying the suggested measures above will be required before the lessons of this review can be applied generally.
